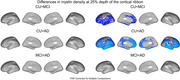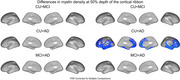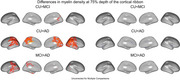# Cortical Myelin Density Patterns Across the Alzheimer's Disease Spectrum

**DOI:** 10.1002/alz70856_097435

**Published:** 2025-12-24

**Authors:** Vincent Koppelmans, Susanne G Mueller, Marit F. L. Ruitenberg, Jace B King, Kevin Duff

**Affiliations:** ^1^ University of Utah, Salt Lake City, UT, USA; ^2^ Department of Radiology, University of California, San Francisco, San Francisco, CA, USA; ^3^ Center for Imaging of Neurodegenerative Diseases, San Francisco, CA, USA; ^4^ Leiden University, Leiden, Zuid‐Holland, Netherlands; ^5^ Leiden Institute for Brain and Cognition, Leiden, Zuid‐Holland, Netherlands; ^6^ Oregon Health & Science University, Portland, OR, USA; ^7^ NIA‐Layton Aging & Alzheimer's Disease Research Center, Portland, OR, USA

## Abstract

**Background:**

Neural degeneration in mild cognitive impairment (MCI) and Alzheimer's disease (AD) is characterized by cortical and subcortical gray matter atrophy. If, and to what extent, this atrophy reflects degeneration of myelinated dendrites is largely unknown. The current study tested differences in myelin density at different cortical depths between cognitively unimpaired (CU) (*n* = 71), MCI (*n* = 51), and AD (*n* = 42) older subjects.

**Method:**

Subjects completed 1mm isotropic MP2RAGE MR imaging. The ratio of the uniform MP2RAGE image and the T1 relaxation map, generated using SPM12's mp2rage toolbox, was projected to the fsaverage template surface using FreeSurfer. Myelin values were projected at 25%, 50%, and 75% depth from the gray matter‐white matter boundary and pial surface to assess myelin levels in different cortical layers. FMRIB Software Library's Permutation Analysis of Linear Models tested for age and sex‐adjusted group differences in cortical myelin using 1000 random permutations and with FDR multiple comparisons correction.

**Result:**

At 25% cortical depth (Figure 1) myelin density in CU < MCI across the left hemisphere. At 25% and 50% depth, myelin density in CU < AD across both hemispheres, except for the medial visual cortex and the central sulcus extending to the primary motor and the somatosensory cortices. At 75% depth (Figure 3), myelin density in CU > MCI > AD in the primary motor, posterior cingulate, superior temporal, and visual cortices, although these latter results did not survive FDR correction.

**Conclusion:**

These results corroborate a study reporting higher cortical myelin density in AD than CU obtained through T1/T2 ratio imaging (Pelkmans et al., 2019). Myelin appears most affected in deeper cortical layers through a process that progresses from MCI to AD. Potential explanations for this counter intuitive finding are compensatory remyelination observed in late stage AD (Bulk et al., 2018), disproportional atrophy of the superficial more stronger myelinated layers in AD thereby shifting the cortical depth lines potentially resulting in the group comparison of different cortical layers (Romito‐DiGiacomo et al., 2007), and that the cortical maps obtained from the MP2RAGE images measure microstructure beyond myeloarchitecture.